# Family's role in long‐term care—A qualitative study of Finnish family members' experiences on supporting the functional ability of an older relative

**DOI:** 10.1111/hsc.13700

**Published:** 2021-12-30

**Authors:** Vilhelmiina Lehto‐Niskala, Outi Jolanki, Marja Jylhä

**Affiliations:** ^1^ Faculty of Social Sciences (Health Sciences) Tampere University Tampere Finland; ^2^ Gerontology Research Center Tampere University University of Jyväskylä Jyväskylä Finland; ^3^ Department of Social Sciences and Philosophy University of Jyväskylä Jyväskylä Finland

**Keywords:** family care, functional ability, long‐term care, nursing homes, older people, residential care

## Abstract

Family members are important providers of care for older people. In residential long‐term care, however, their role is not always simple and straightforward: responsibility for care provision rests officially with staff members, but in practice family members often contribute to providing care. The main reason for admission to long‐term care is functional decline. At the same time, the maintenance of functional ability is a central goal in long‐term care. It is therefore reasonable to assume that functional ability is also an important factor in the relationship between family members and long‐term care residents. This study aims to explore how family members experience their role in supporting the functional ability of older relatives in residential long‐term care. With the approval of the local hospital district's ethics committee, we conducted semi‐structured interviews with family members (*n* = 16) in Finland in 2016. Thematic data analysis showed that family members supported the functional ability of their older parent or spouse by organising and monitoring care and by bringing forth their relative's personal needs and wishes. They often saw their role alongside staff members as ambiguous, and their understanding of the scope of support for functioning extended beyond physical everyday tasks. In their talk, family members broadened the concept of functional ability from daily chores and independence to meaningful social relations and acknowledgement of person's individual background and preferences. Family members’ views offer valuable insights into residents’ personal needs, values and preferences and in doing so help care workers to support their functional ability with a person‐centred care approach.

**What is known about this topic?**

- Family members’ role in the long‐term care of older people is important but ambivalent.
- Supporting and maintaining functional ability of the residents in long‐term care facilities is part of good quality care.

**What this paper adds?**

- Family members considered their role in supporting functioning of their older relative as important.
- Family members valued that their close ones residing in long‐term care facilities received support not just in the everyday physical tasks but in their social and emotional needs and maintaining one's personhood.
- The findings of this study will expand knowledge on how to support functioning of older long‐term care residents.

## 1 INTRODUCTION

Family members have always had an important role in the care of older relatives. Recent changes in care policies only serve to underline the family's caregiving role, especially in countries with a traditionally universalistic care regime (Hoppania, 2018; Szebehely & Meagher, 2018; Van den Broek et al., 2019). Although they do not have a legal obligation to provide care for older relatives, family and friends are expected to assume responsibility for care and support formal care services (Ahosola, 2018; Kalliomaa‐Puha, 2017; Szebehely & Meagher, 2018). The family's caregiving role is often ambivalent (Pillemer et al., 2019), particularly when their older relative lives in long‐term care. While responsibility for care provision officially rests with the formal care system, family members are expected to contribute as well (Milligan, 2009).

Supporting functioning of older people is recognised as one of the main roles of care professionals, (Ministry of Social Affairs & Health, 2018; Vähäkangas et al., 2006) but there are different understandings of how they should go about providing rehabilitative care. For instance, it can be aimed at motivating and encouraging older individuals to perform given tasks as independently as possible (Galik et al., 2014; Hjelle et al., 2017; Resnick et al., 2013, 2014) or completing everyday chores together with the resident (Ministry of Social Affairs & Health, 2018). However, functional ability is an ambiguous term. In World Report on Ageing and Health the World Health Organization defines functional ability as ‘the health‐related attributes that enable people to be and to do what they have reason to value’ (WHO, 2015:28). Functional ability can be viewed as comprising of intrinsic capacity, the environment in its broad meaning and the interaction between individual and the environment (WHO, 2002, 2015). Ageing studies often measure functioning by independence in activities of daily living (ADL) (Chatterji et al., 2015; Corneliusson et al., 2019; Palese et al., 2016). These activities include, for instance bathing, eating and toileting. (Katz et al., 1970) Our previous study showed that for nurses working in long‐term care, functional ability has to do with independence and activities of daily living, whereas residents understand it from a broader perspective in connection with coping and the life course (Lehto et al., 2017). But it is unclear how family members understand functional ability and what role they assume in supporting it.

Many family members continue to look after and support their older relative after the transition to long‐term residential care. Family members often have profound knowledge of their relatives’ routines and preferences. They have an important role as guardians of the identity and dignity of their loved ones (Harnett & Jönson, 2010; Hertzberg & Ekman, 2000; Whitaker, 2009), and can ensure that their relative's previous routines and lifestyles are respected (Davies & Nolan, 2004; Eika et al., 2014; Graneheim et al., 2014; Palmer, 2013; Ryan & Mckenna, 2015). An approach that is increasingly used to answer to the diverse needs of older people with declined functional ability is the concept of person‐centredness. This means that care services are delivered so that they respond to the care receivers’ preferences and personal needs (WHO, 2015). By understanding and knowing the resident thoroughly, family can help to maintain their relative's personhood and support person‐centred care (Kitwood, 1997). However, in long‐term care family members are often regarded as outsiders rather than part of the care community. The transition from home to long‐term care and the process of carving out a new role can be especially difficult when the family member's role changes from principal caregiver to that of a visitor (Crawford et al., 2015; Davies & Nolan, 2004; Graneheim et al., 2014; Ryan & Mckenna, 2015).

This study was conducted in Finland, where long‐term care consists of assisted living with 24‐hr care, nursing homes and long‐term care wards in both hospitals and healthcare centres (Johansson, 2010). The main reason for admission to long‐term care is functional decline. As home care is always the priority option, individuals entering long‐term care usually have several functional limitations. More than half of the long‐term care residents have been diagnosed with memory disorder (National Institute for Health & Welfare, 2021). Maintaining and improving functioning and a rehabilitative approach in care are among the fundamental principles of official care policies, including long‐term care (Ministry of Social Affairs & Health, 2018). The aim of this study was to explore the role of family members in long‐term care, and in particular, their ways to support the functional ability of their older relatives.

## 2 METHODS

This study is part of a research project aimed at finding out how functional ability and rehabilitation are understood in residential long‐term care. The study protocol was approved by the ethics committee of the local hospital district. The data analysed in this study comprise interviews conducted with 16 family members.

Participants were recruited in two municipalities in southern Finland by means of purposive sampling. The eight care facilities included four institutional long‐term care facilities, namely two nursing home wards and two long‐term hospital wards, and four assisted living facilities with 24‐hr care. Assisted living is usually defined as a home because residents will pay a rent and separate fees for care, medication and other additional services. Nursing homes and long‐term care wards are regarded as institutional care where residents pay a single means‐tested fee. In practice, however, assisted living and institutional care address the same care needs, and care practices are rather similar. Two of the facilities were public and six were run privately, with the local authorities having outsourced their services. A more detailed description of the care facilities can be found elsewhere (Lehto et al., 2017). The original reason for choosing a mix of different facilities was to compare different service providers. However, during the analysis we discovered that the experiences of our participants did not differ between different facilities, so they were not separated for the analysis.

The first author contacted the managers of eight care facilities, who were each asked to suggest two family members for a research interview. To be eligible for the study participants’ relative had to have lived in the current facility for at least 6 months. The managers were free to choose any family member they thought was suitable for the study, because they have the best knowledge of residents’ family and how to contact them.

A total of 13 women and three men were interviewed in 2016 (Table 1). The participants had their mother (*n* = 9), father (*n* = 1) or spouse (*n* = 6) living in the facility. The duration of stay ranged from 3 months to 11 years.

**TABLE 1:** Participants in the study

Name	Gender	Age (years)	Kinship	Relative's residence	Duration of relative's residence in current facility
Susanna	Female	71	Daughter	Nursing home	1 year
Riitta	Female	64	Daughter	Nursing home	1 year 4 months
Reijo	Male	Not available	Husband	Nursing home	11 years
Kaarina	Female	61	Daughter	Nursing home	3 months
Pirjo	Female	67	Daughter	Assisted living with 24‐hr care	6 years
Tapani	Male	48	Son	Assisted living with 24‐hr care	3 years
Leena	Female	64	Wife	Assisted living with 24‐hr care	1 year 2 months
Minna	Female	Not available	Daughter	Assisted living with 24‐hr care	1 year
Hannele	Female	76	Wife	Long‐term care hospital ward	2 years 6 months
Anneli	Female	72	Wife	Long‐term care hospital ward	2 years 6 months
Mirja	Female	69	Wife	Long‐term care hospital ward	1 year
Marjatta	Female	64	Daughter	Long‐term care hospital ward	4 years
Tuija	Female	68	Daughter	Assisted living with 24‐hr care	6 years
Niina	Female	63	Daughter	Assisted living with 24‐hr care	3 years
Kari	Male	55	Son	Assisted living with 24‐hr care	2 years
Soile	Female	77	Wife	Assisted living with 24‐hr care	4 years

The semi‐structured one‐on‐one interviews were carried out between August 2016 and November 2016 in the long‐term care facilities where the participants’ relatives lived. They lasted from around half an hour to an hour, were audio‐recorded and transcribed verbatim. The participants were given written and oral information about the study and their right to withdraw at any time. Written informed consents were obtained. The interviews concerned the functional ability of the participants’ older relatives and their rehabilitation in long‐term care. The informants were asked how they would evaluate their relative's health and how they would describe their functional ability, how the care facility maintained their relative's functional ability, what supported and what hindered their functional ability in long‐term care, how they perceived rehabilitation in the current care facility, and who conducted the rehabilitation.

The data were analysed using thematic analysis and the first author conducted the analysis following the guidelines by Braun and Clarke (2006). The first step involved reading and rereading the interview transcripts, focusing especially on how the interviewees explicated their experiences by giving examples of events and activities in which they had been involved. These turns of talk were then coded. Next, coded accounts were collated and sorted into preliminary themes. Analysis continued as an iterative process between the data extracts, the initial themes and the whole data set. At this stage, some themes were merged and renamed while some data extracts were moved to another theme (Braun & Clarke, 2006). Our aim in defining the themes was to grasp the similarities and variation in the participants’ understandings of the topic at hand. To ensure rigorous analysis all three authors discussed the coding and the themes at several stages of the process. The final results were discussed between all authors until consensus was reached.

## 3 FINDINGS

The participants interviewed were asked about their older relative's current health, functional ability and rehabilitation in long‐term care. The interviewer did not explicitly ask them to describe their earlier experiences, yet many chose to illuminate their points of view by describing various events and their experiences about care (or lack thereof), their older parent's or spouse's functional status and their previous experiences from home or from other care facilities.

The themes identified in our analysis were as follows: (1) engaging in daily activities, (2) monitoring care and (3) bringing forth personal needs and wishes. Each main theme included two or three subthemes (Table 2). We illustrate the findings using excerpts from the interviews, originally in Finnish but translated here into English. All names are pseudonyms.

**TABLE 2:** Themes and subthemes identified in the family members’ accounts of maintaining the functional ability of their relative

Theme	Subthemes
Engaging in daily activities	Organising care Providing physical support Taking part in care decisions
Monitoring care	Prior experiences influencing observations of care Filling observed gaps in care
Bringing forth personal needs and wishes	Pointing out their relative's likings Providing meaningful social contacts

### 3.1 Engaging in daily activities

During the interviews, participants often described what they were able to do themselves to support the functional ability of their parent or spouse. Some family members had brought exercise tools or other equipment to the care facility. One participant mentioned the dumbbells that the family had acquired, and one woman said her son had bought audiobooks for her disabled husband. Often the care they offered consisted simply of physical support, such as helping their relative do their exercises:

Every time I visit I try to do my share in helping to walk [my mother] along these corridors. (Susanna)

Because of their close relationship, our participants described themselves as the best persons to motivate their older relatives to do their exercises. Several family members described how they ensured that their relative gets out of bed. Many mentioned their experiences of accompanying the resident outdoors:

It was quite exciting when I took her out into the garden with her new chair. I mean that's a form of rehabilitation too, going outdoors. (Pirjo)

Some family members had organised additional physical therapy to complement what was normally available in the care facility. They were active in arranging activities and acquiring help from outside the care facility:

So now I’ll have to try and find a physiotherapist who you know is sort of oriented towards neurological rehabilitation or you know, could provide it. (Tapani)

Family members supported the functional ability of their relative by taking part in care decisions, saying that they wanted to be involved in care planning. Participants helped with their relative's medication, which in their accounts was associated with supporting functional ability. Especially if they felt their relative had too much or too strong and disabling medication, they would try to inform the care staff and get the physician to reduce the number of drugs:

And then all these changes in medication, I’ve always wanted to be involved. I know what my mother's been taking. I know a bit about these drugs so I want to be involved. (Niina)

When the participants talked about helping their relatives in their daily chores or taking part in planning or organising care, they portrayed themselves as being involved in the everyday care of their relative. From their point of view, this supported the functioning of their relative. Participants stressed the importance of being able to take part in daily activities, but some explained why they could not provide as much care as they wanted to or felt was expected of them. They described how their own health and personal struggles prevented them from taking part in certain activities: they referred, for instance, to their own illnesses and disabilities:

There could be some rehabilitation, it's true. When you have this person who'd want, and who is able, who has possibilities. (Interviewer: Yes, right) And I myself can't, because my rheumatism is so difficult, I don't dare to leave with him. (Soile)

### 3.2 Monitoring care

Many of the participants mentioned an imbalance between their expectations and experiences of care that would support their relatives’ functioning. They often ended up describing and criticising the care received by their family member by contrasting their expectations with the actual care they had observed. From the family members’ point of view, it was important to provide meaningful experiences for their loved ones to maintain their functional abilities. They thought it was the family's role to provide this sense of a meaningful life, something the care facility did not always do. In their opinion, family members had an important role in filling gaps in care:

I mean her functional ability deteriorated from what it was after her stroke. We all understand that when you do nothing but lie down, people won't, when they're not exercised, they'll sort of, get lazy. I’ve now tried to exercise with her, I decided a year ago that I’ll try to do what I can. With my translator's training. (Riitta)

At the same time as they portrayed themselves as filling a gap in care, family members expected care workers to assume greater responsibility for supporting the functional ability of their relatives. However, some family members clearly did not *want* to place demands on staff, even though this meant that they themselves had to carry a greater share of the burden of care. Some pointed out that they did not want to ask staff for help because they felt they were too stretched as it was. Lack of resources was mentioned by many of the participants; Minna explicitly said she did not *‘blame the staff but the lack of resources*’.

Although the participants expressed criticism of the lack of what they perceived as good quality care, some were keen to point out that the care home just did not have the necessary resources to provide the kind of care that would meet their hopes and expectations:

Because she can be aggressive if, no longer but a bit earlier. So, so. The staff like, they can't like, there always has to be two of them. (Interviewer: Right) So you might have this occasional, she might have a swing at them and like. So it hasn't really been very good in the sense that she hasn't been lifted up very often and you know. But I mean I’m sure they don't have the resources either, you know. Because there really aren't a lot of staff members during the evenings and weekends. So sometimes I’ve felt like she's just been left there in the bed. (Marjatta)

Many family members adjusted their expectations according to their earlier experiences of care. Sometimes they described their satisfaction with the care provided in the current facility by reference to their frustration with the care provided in an earlier environment. Moreover, some family members who had had care responsibilities when their relative lived at home and had experienced feelings of insufficiency, talked about the sense of relief they felt when their loved one had been admitted to a care home, even if the care provided was not perfect:

I mean if you consider that he's 86 years old now. That it would be good to cope with what we've got. For years it's been here, I mean here at this level. I did notice that there's been some deterioration but, but, but I mean if we were at home just the two of us, I can't, I can't really offer him these activities in the same way. I mean he has this big bright room and he can move around here and, they take him downstairs, and if there's some musical programme and, erm, erm, and then there's this physiotherapy. We did have a physiotherapist who visited us at home, but they've got all this equipment here and well generally the premises are better. At home it's a bit modest and, that, there are many good sides but, but I do think that if we could more or less maintain the condition in which he's in, as it is. That he could go to the bathroom and get in and out of bed himself. That would be good. (Leena)

Older relatives’ age and illnesses were frequently mentioned in the participants’ evaluations of the care that was aimed at supporting functioning. For these reasons, they felt they had to tone down their expectations. The current status and future functioning of their relative were dependent on factors over which the participants said they had no control. However, some did say they had had experiences of successful rehabilitation and on this basis thought it was indeed possible to see an improvement in functional ability.

### 3.3 Bringing forth personal needs and wishes

In describing their experiences of how their relative's functional ability was supported, family members often began with an account of their older relative's earlier life, referencing their hobbies, interests or occupation. The participants especially described those features and care practices that coincided with the wishes and likings of their relative:

He [her husband] likes going to sauna, and that was a source of great pleasure, and then when he came here, it was all unfamiliar and there were strangers and there was even a black man. And then, erm, then when he'd been here for a while, it was home‐, once in a while I’ll have him home for the weekend. And then he said that this “good black man”, he took him to the sauna and even massaged his neck. So he's now become a good friend, you know. He will, Daniel will take him out and, and, erm, sometimes massage his shoulders and, and it all seems very sympathetic. (Leena)

The activities undertaken or enjoyed by older relatives were mentioned several times in the interviews as having a role in maintaining functional ability.

Family members specifically mentioned individuals in the care community who attended to residents’ personal needs. These kinds of relationships were considered particularly important. The primary nurse was often mentioned in this context, but in one instance reference was also made to a cleaner who regularly talked with one participant's mother. As far as family members were concerned, only few staff members emphasised the personal preferences of their loved one. However, the best way to meet residents’ personal needs, they felt, was in co‐operation with staff:

They've made an effort to find out what my mother likes, the primary nurse here. I mean there was once a note on the door which said ‘I like beautiful clothes’ and again they'd left me a note asking if I wanted to wash the dress myself. And they lacquer her nails and they, I’ve asked them to always put in her hair rollers. (Niina)

Family members were keen to stress their own role in ensuring that their relative could enjoy daily life. They described how the family would incorporate their relative's earlier life preferences into their current life in the care facility. In one of the interviews, a participant called Hannele answered the question about how her husband's functional ability was supported by listing a string of items (e.g. sufficient nutrition, personal hygiene, pedalling an exercise bike, participating in events at the care home). But then she continued:

And then there's this mental side that we try to keep up, keep up by getting him involved in all the events they have here. And somehow to get, like get his mood, his mood in the right place. Like for instance we have out in the country, we have two farms. Or one farm and one sort of cottage. So my son's set up these cameras. And then he's put this kind of receiver, which is connected to the tv in his room. So that he can sit in his room or lie in bed and follow what's happening there. (Hannele)

Maintaining social contacts was considered an important part of functional ability by many family members. They portrayed themselves as important providers of social interaction. Participants frequently described how they supported their relative's social functioning, or in the words of Kaarina, how they brought *‘something positive into their life*’. Some family members regularly took their relatives to visit their homes or other familiar surroundings. Often the family member's visit to the care facility was in itself portrayed as a way of supporting social functioning.

In several interviews, family members described themselves as sources of emotional support. This was accepted as part of their role as a family member. However, sometimes our participants portrayed their support as a matter of necessity:

My understanding is that rehabilitation is something that, like you see an improvement in a person's state of being. An improvement in functioning, in mood, you see something positive in life, that life is worth living, or something like that. [text omitted] For example when my daughter, daughter came here to visit here with her little son and it was just at the time when he was starting to smile and look around, my mother still remembers it. She didn't forget. That the boy laughed or that the boy smiled at her. So these kinds of things stay with her, and they'd be important to have. And of course I do hope that here on this ward, that it wouldn't totally depend on me and my daughter, what happens to her during the day. (Kaarina)

Kaarina implies that rehabilitation should include emotional support, and that currently that support only comes from the family. While some accepted this role, many suggested that care staff should pay more attention to providing emotional support to their relative.

Social contacts and social interaction where the persons’ subjective needs and likings were taken into account were regarded by our participants as important to supporting functional ability. Family members had an important role to play in providing news and social contacts from outside the institution. They were the link between the care institution and the individual resident as part of his or her family and with his or her individual background, personality and wishes.

## 4 DISCUSSION

This study explored how family members support and maintain the functional ability of their older relatives in residential long‐term care. Supporting functional ability is considered one of the main responsibilities of professional care staff (Ministry of Social Affairs & Health, 2018; Vähäkangas et al., 2006). However, according to the results of this study also family members were actively involved in providing care that supported the functional ability of their loved ones. They participated in physical exercise, organised and monitored care and ensured that their older relatives’ social and emotional needs were met.

Long‐term care is widely premised on a rehabilitative approach (Galik et al., 2014; Resnick et al., 2013, 2014; Vähäkangas et al., 2006). Therefore, the views of family members on supporting functioning are likely to shed light on their role in long‐term care more generally. However, the role of family members in this context and their contribution to supporting the functioning of older relatives has so far received scant attention. While the role of family members and their support in different care settings is widely acknowledged (Ågård & Lomborg, 2010; Oyesanya & Bowers, 2017), research has shown that it can be challenging for family members to find their place in the formal care environment (Crawford et al., 2015; Graneheim et al., 2014; Ryan & Mckenna, 2015). Our study showed that family members were engaged in the everyday life of the care home and participated in supporting the functioning of their parent or spouse. The majority described their involvement and participation in various activities as a matter of necessity. A sense of dissatisfaction and disappointment about care quality led to family members assuming greater responsibility for care provision themselves and filling the gaps in care.

Functional ability and rehabilitative care occupy a prominent place in long‐term care policies (Ministry of Social Affairs & Health, 2018). To support residents’ functional ability, care professionals are expected to encourage residents to perform tasks independently (Galik et al., 2014; Hjelle et al., 2017; Resnick et al., 2013, 2014) or to complete daily chores together with them (Ministry of Social Affairs & Health, 2018). In our study, family members assumed partly different role: they also valued support for social and emotional needs (Milligan, 2009; Palmer, 2013). Our findings support, but also add to earlier studies which suggest that the family has an important role in ensuring that the resident's earlier routines and preferences are respected (Davies & Nolan, 2004; Eika et al., 2014; Graneheim et al., 2014; Ryan & Mckenna, 2015). In doing so, family members assumed responsibility for maintaining their relative's personhood, the intrinsic uniqueness of a person that is recognised and maintained in social interaction (Buron, 2008; Kitwood, 1997; Manthorpe & Samsi, 2016). Our results show that maintaining personhood is crucial in supporting the functioning of older persons in long‐term care, and that family members can help to achieve this goal by providing meaningful social contacts and informing the care staff about their relative's wishes and likings. Family members’ views were consistent with a person‐centred care approach that underscores the importance of considering the care recipient's personal needs and preferences (WHO, 2015).

In the context of ageing research, functional ability is often understood in terms of activities of daily living (Chatterji et al., 2015; Corneliusson et al., 2019; Palese et al., 2016). Our previous study indicated that staff members often describe functional ability as abstract domains or tasks of daily living, while older residents themselves also talked about activities that were important to their quality of life, such as painting or watching TV (Lehto et al., 2017). The present study indicates that for the family, personal preferences, individual background and meaningful social relations are crucial elements of functional ability. The views of family members lend support to the understanding of functional ability as consisting of both individual and environmental factors, which include relationships, values and care services (WHO, 2002; WHO, 2015).

It is a matter of ongoing debate whether family members should contribute to long‐term care provision or whether they are regarded merely as visitors (Crawford et al., 2015; Hertzberg & Ekman, 2000; Ryan & Mckenna, 2015). In fact, family members are important contributors to care because of their close, ongoing relationship with their loved ones. We argue that family members could be even more closely involved as partners in care so that residents’ functioning can be better maintained. At the same time, this would contribute to widen the understanding of functional ability in long‐term care.

The participants in this study were recruited through the care managers of the care facilities concerned. It became evident during the interviews that all the participants took an active part in care provision. Moreover, several of them indicated that they had to some extent been involved in taking care of the resident even before their placement in long‐term care. It is possible that the family members the managers recommended for research interviews were particularly active visitors at the care facility and well‐known by staff members—and therefore that there are other family members who are less actively involved in the care of their relative and whose experiences might well differ from those who were interviewed in this study. In addition, our study has not addressed the situation of residents who have no family members, even though, Ahosola (2018) recently pointed out that more attention should be given to the functional ability and the social and emotional needs of those residents without a family (Ahosola, 2018). However, we believe that our family members’ detailed accounts of their experiences of functional ability and rehabilitation in long‐term care provide important knowledge for the development of long‐term care practices. The results of this study show that family members have an important role in supporting residents’ functional ability in long‐term care, but also in expanding the meaning of functional ability in a way that further emphasises the importance of person‐centred care.

## 5 CONCLUSIONS

Our results show that family members consider themselves to have an important role in supporting the functional ability of their older relatives living in long‐term care. Their talk implies that as care homes aim to maintain the functioning of their residents, functional ability should be understood in broader terms than just daily chores and independence. Holistic support for functioning means that the person's individual background and preferences are respected and that maintaining his or her personhood is understood as an important part of maintaining functional ability. Here, family members should be seen as partners in care and given the opportunity to participate in the care of their close ones if and when they want to do that. At the very least family members’ views offer valuable insights into residents’ personal needs, values and preferences and in doing so help care workers to support their functional ability with a person‐centred care approach.

## CONFLICT OF INTEREST

We declare no conflict of interest.

## Data Availability

Research data are not shared due to privacy and ethical restrictions.
